# Editorial: Models of Reference

**DOI:** 10.3389/fpsyg.2016.01855

**Published:** 2016-12-05

**Authors:** Kees van Deemter, Emiel Krahmer, Albert Gatt, Roger P. G. van Gompel

**Affiliations:** ^1^Department of Computing Science, University of AberdeenAberdeen, UK; ^2^Tilburg Centre for Cognition and Communication, Tilburg UniversityTilburg, Netherlands; ^3^Institute of Linguistics, University of MaltaMsida, Malta; ^4^School of Social Sciences, University of DundeeDundee, UK

**Keywords:** psycholinguistics, language production, referring expressions, computational models, audience design

## 1. Introduction

To communicate, speakers need to make it clear what they are talking about. *Referring expressions* play a crucial part in achieving this, by anchoring utterances to things. Examples of referring expressions include noun phrases such as “this phenomenon,” “it,” and “the phenomenon to which this Topic is devoted.” Reference is studied throughout the Cognitive Sciences (van Deemter, 2016).

Recent years have seen a new wave of work in this area, as witnessed by a number of journal Special Issues.^1^The Research Topic “Models of Reference” in *Frontiers in Psychology* is a new milestone, focussing on contributions from Psycholinguistics and Computational Linguistics.

Unsurprisingly given the journal, the response to our Call for Papers has focussed predominantly on psycholinguistic work. A majority of submissions dealt with language production, as opposed to comprehension. In what follows, we summarize the papers accepted for this Research Topic, stressing some of the main themes emerging, including audience design (Section 2); overspecification (Section 3); visual perception, and variation between speakers (Section 4). We end with some general observations.

## 2. Audience design and theory of mind use

Successful communication requires that speakers and hearers take each other's knowledge into account, yet recent studies have questioned the extent to which they are able to do this (e.g., Horton and Keysar, 1996; Keysar et al., 2003). The present Research Topic shows that reference is still a key battleground in this debate.

Ibarra and Tanenhaus, for instance, investigate to what extent interlocutors are able to switch between different ways to conceptualize an object, as a function of the conversational setting in which the dialogue takes place; for example, a part of an object may be called a “wrench” in one setting (because it looks like one) but a “leg” in another (because that's its function). The authors conclude that switching between conceptualizations takes place with remarkable ease: “conceptual pacts are fluid temporary agreements.” Branigan et al. focus on 8- to 10-year-old children, investigating the extent to which these are able to assess Common Ground developed through prior linguistic context, and whether this is sensitive to variations in prior interactions with the listener. Similar to adults, children adjust the length of their referring expressions depending on whether, in the preceding conversation, their conversational partner was a side-participant (who both saw the object and heard the expression), an overhearer (who only heard the expression), or a new participant. Nadig et al. put the spotlight on adults with Autism Spectrum Disorder. Although these were less likely than neurotypical speakers to adapt their referential behavior to their interlocutor, what stands out from their work, is how subtle the differences in behavior were, given that referential Audience Design may be thought to be particularly challenging for people with autism (see also earlier studies such as Begeer et al., 2010).

A new strand of work seeks to model interlocutors' reasoning about Common Ground more precisely than before. Our Topic contains two examples, which complement each other neatly: The article by Gegg-Harrison and Tanenhaus follows on from Heller et al. (2012), asking what an interlocutor can figure out, from earlier dialogue moves, about the likelihood that her interlocutor is familiar with a given proper name. Kutlák, et al. focus on situations where the hearer does not know the name of the referent, so it is crucial that suitable properties are selected for inclusion in the referring expression. The authors offer a computational model of a speaker's assessment of the likelihood that the hearer knows about any given property of a referent (e.g., that Darwin wrote “On the Origin of Species”).

Horton and Brennan, finally, step back to discuss the information stored by interlocutors about what has been said over the course of a dialogue, asking what it is that they remember of it, and whether these meta-representations are subject to specific constraints. Proposing a synthesis of their earlier work, they argue that “any representations that capture information about others' perspectives are likely to be relatively simple and subject to the same kinds of constraints on attention and memory that influence other kinds of cognitive representations.” The use of generic psychological mechanisms is a theme that can be discerned in other papers as well, as we will see.

## 3. Overspecification

Much research has focussed on speakers' inclination to include more information in referring expressions than hearers require for identifying the referent (Pechmann, 1989; Engelhardt et al., 2006), a phenomenon known as overspecification. Overspecification features strongly in the contributions by Rubio-Fernández, by Tarenskeen et al., and by Westerbeek et al.

Westerbeek et al. examine an idea that, though it has been examined in the past (e.g, Sedivy, 2003), has recently come to the fore, namely that properties that are *atypical* for a particular type of object are particularly likely to be included in a reference to the object. While these authors confirm earlier findings, focussing especially on color, they also find that the color typicality effect is moderated by color diagnosticity: it is strongest for high-color-diagnostic objects (i.e., objects with a simple shape). Atypicality is likewise discussed by Rubio-Fernández. Scrutinizing the well-attested propensity of color terms to be used in overspecification, she found that this propensity is modulated by factors such as typicality and the extent to which color can facilitate object recognition. Tarenskeen et al. focus their take on these issues on another dimension of variation between properties, namely whether they express an absolute property (such as color) or a relative one (such as size).

The contribution of Brodbeck et al. shows, following on from earlier studies such as Engelhardt et al. (2011), how brain studies that measure erp can track the time course of the process whereby a hearer comprehends a referring expression. Among other things, their work suggests that when we read an overspecified expression, then even after we have identified the referent, we *reactivate* the corresponding representation when processing additional words. The authors argue that this might explain the benefits that overspecification (cf. Section 3) has been shown to have in some situations.

Finally, while the paper by Pogue et al. is concerned with overspecification, it is also relevant to our proposed theme of rationality. These authors asked how listeners might make rational use of linguistic information despite the fact that the linguistic input to which they are exposed often includes more, or less, information than what is necessary and sufficient for a given referential intention. Their model suggests that part of the answer lies in hearers' ability to adapt their expectations to a particular speaker. This brings us to a final strand of work discussed in this Topic.

## 4. Vision and individual variation

### 4.1. Visual perception and salience

Reference, of course, is not tied to any particular perceptual modality—we routinely talk about things we have never seen. Yet much of our knowledge of the production of referring expressions has focussed on visual domains. A number of articles in this Topic extend this body of knowledge, with an emphasis on links with visual perception.

The papers by Clarke et al. and by Baltaretu et al. exemplify this line of work, inquiring how the perceptual configuration of a visual display influences reference. Baltaretu et al. find that when referring to an object using a spatial relation (e.g., “the ball between the doll and the train”), speakers' choice of relatum depends in part on its spatial location in the scene. Clarke et al. focus on scenes with visual clutter. Their main finding is that the visual salience of objects affects their order of mention in a description, a finding that is mirrored by an experiment in which salient objects are shown to be detected faster if they are mentioned earlier. Taken together, these findings support the view that visual perception is tightly coupled with language use (Tanenhaus et al., 1995).

### 4.2. Individual variation

Variation between people is a basic observation in psychology, and studies of language production are starting to focus on this reality. Using machine learning, Kibrik et al. offer a model of the choice between different types of referring expression. They focus directly on the issue of variation, examining its implications for computational models of language production. The contribution by Hendriks takes a more theoretical approach, hypothesizing that differences in cognitive capacity can explain an important part of the observed variation between speakers. Hendriks discusses a model based on the cognitive architecture ACT-R (e.g., Anderson, 1993), which focusses on individual differences in processing speed and working memory capacity, arguing that these factors can be predictive of both underspecification and overspecification, and of listeners's tendency to misunderstand referring expressions as well. The contribution by Peters et al., finally, argues that although pronouns and repeated references are processed in different ways, these differences can be explained by general memory principles such as interference, suppression, and competition. This idea is consonant with those of Horton and Brennan (see above), who emphasize generic psychological mechanisms as well.

## 5. Conclusion

Collectively, the papers in this Research Topic show that the study of reference is continuing to attract a large amount of interesting work. Speaking in general, we were struck by an openness to new research methods and paradigms, including neuro-cognitive methods and computational modeling.

Focussing more specifically on the aforementioned themes, we continue to see a large amount of work on **audience design**, but rather than investigating whether adults are cooperative or not (as in most previous research), researchers now realize that it is not an all-or-nothing issue and investigate what information speakers use for being cooperative, they study different participant populations and build models of cooperative behavior in a range of different communicative situations. As for **overspecification**, where earlier work has tended to single out particular properties (e.g., color) as having a high propensity for being used in overspecification, the papers in this Topic paint a subtler picture, where a property may be overspecification-prone in some situations but not in others. Research on **individual variation**, finally, is still in its infancy, but the paper by Hendriks shows one promising direction in which this research may go, by focusing on general memory principles and known cognitive differences between individuals. We expect that these issues will be fleshed out in future by new computational models as well as by brain studies.

## Author contributions

All authors listed have made substantial, direct and intellectual contribution to the work, and approved it for publication.

## Funding

The first author acknowledges support from the EPSRC for the project RefNet (An interdisciplinary Network Focussing on Reference), EP/J019615/1.

### Conflict of interest statement

The authors declare that the research was conducted in the absence of any commercial or financial relationships that could be construed as a potential conflict of interest.

## Footnotes

^1^For instance, in *Language, Cognition, and Neuroscience* in 2014 (http://www.tandfonline.com/toc/plcp21/29/8), and in *Topics in Cognitive Science* in 2012 (http://onlinelibrary.wiley.com/doi/10.1111/tops.2012.4.issue-2/issuetoc. See also Gatt et al. (2014).

## References

[B1] Anderson J. R. (1993). Rules of the Mind. Hillsdale, NJ: Lawrence Erlbaum Associates.

[B2] Begeer S. Malle B. Nieuwland M. S. Keysar B. (2010). Using theory of mind to represent and take part in social interactions: comparing individuals with high-functioning autism and typically developing controls. Eur. J. Dev. Psychol. 7, 104–122. 10.1080/17405620903024263

[B3] Engelhardt P. Bailey K. G. Ferreira F. (2006). Do speakers and listeners observe the Gricean Maxim of Quantity? J. Mem. Lang. 54, 554–573. 10.1016/j.jml.2005.12.009

[B4] Engelhardt P. E. Demiral S. B. Ferreira F. (2011). Over-specified referring expressions impair comprehension: an ERP study. Brain Cogn. 77, 304–314. 10.1016/j.bandc.2011.07.004 21840639

[B5] Gatt A. Krahmer E. Van Deemter K. Van Gompel R. (2014). Models and empirical data for the production of referring expressions. Lang. Cogn. Neurosci. 29, 899–911. 10.1080/23273798.2014.933242

[B6] Heller D. Skovbroten K. Tanenhaus M. (2012). To name or to describe: shared knowledge affects referential form. Top. Cogn. Sci. 4, 166–183. 10.1111/j.1756-8765.2012.01182.x 22389094 PMC3811076

[B7] Horton W. S. Keysar B. (1996). When do speakers take into account common ground? Cognition 59, 91–117. 10.1016/0010-0277(96)81418-1 8857472

[B8] Keysar B. Lin S. Barr D. J. (2003). Limits on theory of mind use in adults. Cognition 89, 25–41. 10.1016/S0010-0277(03)00064-7 12893123

[B9] Pechmann T. (1989). Incremental speech production and referential overspecification. Linguistics 27, 98–110. Incremental speech production and referential overspecification.

[B10] Sedivy J. (2003). Pragmatic versus form–based accounts of referential contrast: evidence for effects of informativity expectations. J. Psycholinguist. Res. 32, 3–23. 10.1023/A:1021928914454 12647560

[B11] Tanenhaus M. K. Spivey-Knowlton M. J. Eberhard K. M. Sedivy J. G. (1995). Integration of visual and linguistic information in spoken language comprehension. Science 268, 1632–1634. 10.1126/science.7777863 7777863

[B12] van Deemter K. (2016). Computational Models of Referring: A Study in Cognitive Science. Cambridge, MA: MIT Press.

